# Physiological requirements for iron in women of reproductive age assessed by the stable isotope tracer technique

**DOI:** 10.1186/s12986-019-0384-1

**Published:** 2019-08-19

**Authors:** Jiaxi Lu, Jie Cai, Tongxiang Ren, Jinghuan Wu, Deqian Mao, Weidong Li, Yu Zhang, Jianhua Piao, Jun Wang, Lichen Yang, Xiaoguang Yang, Yuxia Ma

**Affiliations:** 1 The Key Laboratory of Trace Element Nutrition of The Ministry of Health, National Institute for Nutrition and Health, Chinese center for disease control and prevention, 29 Nan Wei Road, Xicheng District, Beijing, 100050 People’s Republic of China; 2 Hospital Management Institute, Zhongnan Hospital of Wuhan University, 169 Donghu Road, Wuchang District, Wuhan, Hubei 430071 People’s Republic of China; 3 National Institute of Metrology, National Research Center for Certified Reference Material, No.18, Bei San Huan Dong Lu, Chaoyang District, Beijing, 10050 People’s Republic of China; 4 Hebei Medical University, 361 Zhongshan East Road, Shijiazhuang, Hebei 050017 People’s Republic of China

**Keywords:** Iron, Physiological requirement, Women of reproductive age, Stable isotope tracer technique

## Abstract

**Background:**

Iron nutrition is important for the health of women of reproductive age, and defining the physiologic requirement for iron can help them accurately plan the iron intake. However, research on the physiologic requirement for iron in women is insufficient worldwide. This study aimed to further improve the methodology and get more precise data for the physiological requirements for iron in women of reproductive age on the basis of our previous study.

**Method:**

Sixty-one women of reproductive age who had not been pregnant before and during the whole study were included from Hebei province, China in 2015. Each subject participated in a 2-week metabolic trial with consuming 50 mg of the stable isotope ^58^Fe, and were then followed for ~ 800 days. The abundance of ^58^Fe and the total iron concentration in the circulation were measured using multi-collector inductively-coupled plasma mass spectrometry and atomic absorption spectroscopy. The physiologic requirement for iron in women of reproductive age was then calculated.

**Results:**

The average iron circulation rate was 80.4%, and the steady period started from about 1 year. The average physiological requirement for iron of 21 subjects obtained by formula calculation was 1.55 mg/d and 23.63 μg.kg^− 1^.d^− 1^ after adjustment for body mass, and that of 33 subjects obtained by linear regression was 1.29 mg/d, 20.98 μg.kg^− 1^.d^− 1^ after adjustment for body mass. The results by two methods showed no significant difference. The EAR and RNI calculated from this results was 11–13 mg/d and 15–18 mg/d, respectively, both of which were slightly lower than the recommended value in Chinese Dietary Reference Intake (2013).

**Conclusion:**

The physiological requirements for iron in women of reproductive age were in accordance with other studies, while the EAR and RNI calculated from which were slightly lower than Chinese present recommended value.

**Trial registration:**

ChiCTR, ChiCTR-OCH-14004302. Registered 14 February 2014, http://www.chictr.org.cn/enindex.aspx

**Electronic supplementary material:**

The online version of this article (10.1186/s12986-019-0384-1) contains supplementary material, which is available to authorized users.

## Background

Iron is an essential trace element to human health, especially for women, who are more vulnerable to iron deficiency anemia (IDA) because of menstruation and pregnancy [1]. The global prevalence of anemia in 2010 was 32.9%, which lead to 68.36 million years lived with disability, accounting 8.8% of total for all conditions, and half of these caused by IDA [2]. The Chinese national nutrition survey also showed that the total anemia prevalence in China was 9.7% in 2010–2012 years, which was 12.6% in women and 15% in women of reproductive age [3]. IDA can lead to a series of health hazards, such as infection [4], heart failure [5], low birth weight in newborns [6], and increase the mortality risk of parturients and newborns [7, 8]. In addition, the upper intake levels (UL) of iron is low and iron overload may lead to various pathological clinical outcomes, such as pancreatic damage, cardiovascular disease, neurological disease and cancer [9–11]. Therefore, the suitable iron intake is important for women who are in reproductive age.

To take in iron reasonably, accurate dietary reference intakes (DRIs) are critical. In order to calculate the DRIs exactly, we must firstly obtain accurate physiological requirements, which is the core component of DRIs. Evidence shows that iron balance in humans is dependent on the variations of absorption and excretion from the body [12, 13]. The iron physiological requirement in adult males who have no significant change in weight is usually regarded equal to iron loss [14]. The classical research methods for iron physiological requirements, including metabolic balance method and isotope tracer method, often study iron requirements through iron loss. Metabolic balance method collects different ways of iron loss to get the total iron loss, while its result may sometimes be not accurate due to the overestimated or underestimated iron absorption rate [15]. The isotope tracer technique is accurate, and highly sensitive, while it need to be tracked for a relatively long period of time. Since 1939, a series studies on physiological requirements for iron were carried out, using radioisotopes ^55^Fe or ^59^Fe [16–18]. Although the radioactivity of radioisotopes could be accurately tested by the detector, which is convenient and feasible, its potential health hazards and ethical problems limited its application in current researches. In 2005, Fomon et.al began to use stable isotope ^58^Fe to research the physiological requirements for iron in toddlers [19]. In 2018, our team also used ^58^Fe to study the physiological requirements for iron in young men and women with formula calculation method [20]. However, the direct data for women are still insufficient. Among all these published studies, only two of them involved women, which were reported by Finch et al.in 1959 (*n* = 18) and by our team in 2018 (*n* = 7) [20, 21]. In this study, we had used stale isotope ^58^Fe to detect the physiological requirements for iron in women of reproductive age and compared the formula calculation method and linear regression method when processing data.

## Methods

### Subjects and experimental design

82 women of reproductive age were recruited in 2 groups in Xingtang County, Hebei province in January 2015 and March 2015 respectively. 61 of them had not been pregnant during the whole study and were included in this article. Participants were enrolled if they were aged 20–35 years and planned to become pregnant in the near future. Potential participants were excluded if they: 1. had a disease that could affect iron absorption or metabolism (such as malabsorption, gastrointestinal ulcer, or inflammatory disease), or had abnormal iron nutritional status; 2. regularly took medication that could affect iron absorption or metabolism; or 3. they were already pregnant or would be in menstruation during the metabolic trial.

After basic information collection, 2 groups of subjects participated in the metabolic trials in January 2015 and March 2015, respectively. At the beginning of the trial, subjects consumed ^58^Fe in meals in the form of ^58^FeSO_4_, 10 times over consecutive 5 days, ~ 50 mg totally. During this 2-week period the participants stayed in arranged accommodation to ensure compliance with the regimen. Diets and stool samples were collected to get the absorption of iron. Blood sample of each participant was designed to be collected for measuring on the first month, second month, forth month and then every 4 months after the ending of the trial. Participants who had not been pregnant during the whole study would be included in the cohort of women of productive age and followed for more than 2 years. Subjects who got pregnant and their infants were also followed, but their data are not reported here. Throughout the follow-up, attention was paid to the health condition of all subjects to ensure that there were no traumatic blood loss and abnormal iron metabolism. The trials were exactly the same designed in two groups, except when the trial began and the blood samples were collected. The flow chart of study was Additional file 1: Figure S1.

The trial was approved by the Ethics Committee of the National Institute of Nutrition and Health, Chinese Centers for Disease Control and Prevention and registered at the Chinese Clinical Trial Registry (No: ChiCTR-OCH-14004302). Written informed consent was obtained from all subjects prior to their participation.

### Sample analysis

The blood samples were acid digested using a Microwave Digestion System (Mars 6, GEM, USA) with 70% HNO_3_ solution before measuring. The digestion procedure was as follows: 120 °C: ramp 6 min, hold 5 min; 150 °C: ramp 5 min, hold 15 min; 190 °C: ramp 5 min, hold 30 min; 1600 W. Total iron concentration was quantified using atomic absorption spectroscopy (AAS) (PinAAcle 900, PerkinElmer). The abundance of ^58^Fe was analyzed using the Multi-collector inductively-coupled plasma mass spectrometry (MC-ICP-MS) with a standard-sample bracketing method. A mixture of argon and H_2_ were used as collision gas to eliminate the interferences [22]. Under optimized conditions, the precision was 0.01–0.03% (relative standard deviation, RSD). Iron biochemical indexes were measured by the automatic iron biochemical analyzer (Hitach7180, Japan), including serum ferritin (SF), unsaturated iron-binding capacity (UIBC), serum iron (SI), transferrin (TRF), inflammation markers of C-reactive protein (CRP), and α- acid glycoprotein (α-AGP).

### Calculation method

Two methods, formula calculation method and linear regression method, were used to calculate the physiological requirements for iron and were compared. The formula calculation method was based on the change in abundance of ^58^Fe during a set period of time, and the calculation of the mean iron requirement across various time periods. The formula was derived in our previously published work [20]. After the iron isotopes ^58^Fe taken in test days were completely mixed with the iron in the body of the subjects and reached a steady state, the daily loss of iron was calculated by the change in iron isotope during a period of time (assuming day i to day i + t).

1 $$ \mathrm{R}=\mathrm{T}\times \mathrm{V}\times \left({\mathrm{P}}_{\mathrm{i}}-{\mathrm{P}}_{\mathrm{i}+\mathrm{t}}\right)\div \mathrm{t}\div \left[\left({\mathrm{P}}_{\mathrm{i}}+{\mathrm{P}}_{\mathrm{i}+\mathrm{t}}\right)/2-\mathrm{NA}\right]\div \mathrm{C} $$

The linear regression method was based on the study by Fomon et al. in 2005 [19]. Since the ^58^Fe abundance changed exponentially in the body, the original value of abundance was logarithmic to analyze by regression. The slope k was the percentage of iron loss per unit time. The loss of iron in unit time could be obtained by the total iron content in body and the slope.

2 $$ \mathrm{R}={\mathrm{Fe}}_{\mathrm{loss}}=\mathrm{k}\times \mathrm{T}\times \mathrm{V}/\mathrm{C} $$

T: the concentration of total iron in the blood (mg/L).

Pi: the isotopic abundance on day i.

NA: the natural abundance of isotopes.

t: total number of days in the period of time for calculation.

R: daily loss or intake of iron (mg).

V: blood volume (L), which was by the formula published by Carlsen and Bruun [23], V (ml) = (45.2 + 25.3 × exp.(− 0.0198 × DDW)) × BW (kg), where DDW = 100 × (BW (kg) − 7.582 × exp.(0.01309 × BH (cm))) / 7.090(0.01309 × BH (cm)).

C: the iron circulation rate (the proportion of iron in the blood to total iron in the body).

The iron circulation rate was calculated based the method by Fomon in 2005 [19]. Total body iron (Fe_tot_) was consisted of 3 compartments, circulating iron (Fe_circ_), non-circulating active iron (Fe_nca_), and storage iron (Fe_stor_).

3 $$ \mathrm{C}={\mathrm{Fe}}_{\mathrm{circ}}\div \left({\mathrm{Fe}}_{\mathrm{circ}}+{\mathrm{Fe}}_{\mathrm{nca}}+{\mathrm{Fe}}_{\mathrm{stor}}\right) $$

Based on the study by Fomon, Fe_nca_ in body is 6 mg/kg, Fe_stor_ (μg) = 9380 × lgSF-11,260 [24]. Different from using hemoglobin to estimate Fe_circ_, we used the measured concentration of total iron in the blood and blood volume to calculate.

### Statistical analysis

Statistical analysis was performed using SAS 9.4. The normality of the data distribution was investigated with the Kolmogorov-Smirnoff test. Variables that conform to normal distribution were expressed as mean ± SD, and others were expressed as median (lower quartile, upper quartile). Differences between two groups were evaluated using Student’s *t*-test and among more than two groups using Analysis of Variance (ANOVA). The time when the change of isotopic abundance became steady was determined by the Repeated Measures Analysis of Variance Analysis (RMANOVA) combined with the line chart. The rank sum test was used to compare CRP and sTfR values on different days. *P* < 0.05 was considered to represent statistical significance.

## Results

### Following-up condition

Eighty-two women of reproductive age were recruited, and 61 of them who had not get pregnant during the study were included in this study. Their average age was 29.26 y, average height was 159.03 cm, and average weight was 63.76 kg. The hemoglobin and ferritin levels of all subjects were in the normal range. Venous blood samples in group 1 were collected on day 0, 19, 36, 125, 248, 386, 503, 629, 739, 880, respectively. Venous blood samples in group 2 were collected on day 0, 21, 39, 130, 253, 376, 499, 630, 810, respectively. However, with the progress of following up, some subjects dropped out of the cohort for the reason of unwillingness to continue or loss of connection. The number of samples collected in 2 groups was 61 on the first collection (day 0, when the trial started), 48 on the second colletion (~ day 20) and 46 on the 6th collection (~ 1 year).

The indexes of iron nutritional status, including SF, UIBC, SI, TRF, CRP and α-AGP, of subjects were detected at different collection time. The results of these indexes were all in the normal range, which meant that the iron nutrition status of all subjects remained normal throughout the follow-up period, without extra iron requirement or loss. The detail iron nutrition indexes results and following up condition are described in Tables 1 and 2.

**Table 1:** Iron nutritional status in group 1

day	n	SF (μg/L)	UIBC (μmol/L)	SI (μmol/L)	TRF (g/L)	CRP (mg/L)	α-AGP (g/L)
0	23	18.23 ± 13.92	38.41 ± 11.31	10.49 ± 6.37	2.20 (1.92,2.54)	0.40 (0.2,1.1)	0.80 ± 0.22
19	10	14.33 ± 9.92	55.56 ± 14.69	11.56 ± 9.08	3.25 (2.94,3.51)	0.35 (0.2,0.4)	0.48 ± 0.12
36	15	24.43 ± 16.00	50.18 ± 14.63	15.71 ± 7.54	3.10 (2.84,3.39)	0.65 (0.2,1)	0.56 ± 0.15
125	14	17.50 ± 10.69	41.00 ± 18.01	11.81 ± 6.20	2.68 (1.61,3.15)	0.5 (0.3,0.9)	0.60 ± 0.13
248	16	22.44 ± 21.29	39.56 ± 15.87	9.92 ± 5.37	2.19 (1.81,2.84)	0.55 (0.25,1.3)	0.69 ± 0.16
386	14	27.86 ± 27.96	32.43 ± 15.55	9.02 ± 3.86	2.05 (1.81,2.51)	0.35 (0.2,1.5)	0.77 ± 0.17
503	11	25.73 ± 22.62	36.00 ± 12.88	7.59 ± 3.55	2.15 (1.75,2.55)	0.8 (0.2,2.5)	0.76 ± 0.23
629	11	26.36 ± 26.04	27.82 ± 11.84	10.54 ± 5.77	1.90 (1.56,2.41)	0.4 (0.2,1.9)	0.73 ± 0.20
739	12	21.42 ± 17.79	33.58 ± 12.11	10.65 ± 6.67	2.29 (2.04,2.63)	0.35 (0.15,0.75)	0.69 ± 0.19
880	9	28.00 ± 24.37	42.11 ± 14.84	9.33 ± 5.13	2.50 (2.14,3.03)	0.5 (0.4,1.9)	0.72 ± 0.27

*SF* serum ferritin, *UIBC* unsaturated iron-binding capacity, *SI* serum iron, *TRF* transferrin, *CRP* C-reactive protein, *α-AGP* α- acid glycoprotein

**Table 2:** Iron nutritional status in group 2

day	n	SF (μg/L)	UIBC (μmol/L)	SI (μmol/L)	TRF (g/L)	CRP (mg/L)	α-AGP (g/L)
0	38	23.89 ± 16.59	38.73 ± 15.15	14.75 ± 7.51	2.47 (2.20,2.86)	0.3 (0.1,0.5)	0.63 ± 0.21
21	38	21.71 ± 16.61	33.46 ± 11.3	11.79 ± 6.12	2.09 (1.91,2.46)	0.2 (0.1,0.6)	0.67 ± 0.21
39	35	17.77 ± 12.15	34.37 ± 11.95	11.62 ± 5.27	2.13 (1.79,2.48)	0.3 (0.1,0.7)	0.61 ± 0.19
130	33	17.97 ± 13.21	36.43 ± 12.3	12.22 ± 5.25	2.30 (2.00,2.63)	0.2 (0.1,0.2)	0.56 ± 0.15
253	30	18.70 ± 20.14	34.13 ± 9.82	9.92 ± 5.84	2.06 (1.76,2.55)	0.25 (0.1,0.4)	0.63 ± 0.22
376	32	30.93 ± 22.13	24.42 ± 7.26	8.74 ± 5.31	1.58 (1.44,1.83)	0.2 (0.1,0.3)	0.78 ± 0.24
499	33	28.80 ± 29.68	30.65 ± 10.44	8.46 ± 4.81	1.88 (1.45,2.27)	0.2 (0.1,0.4)	0.65 ± 0.20
630	31	25.00 ± 23.35	42.68 ± 16.81	11.76 ± 7.39	2.47 (2.19,3.37)	0.2 (0.1,0.4)	0.66 ± 0.22
810	27	20.38 ± 16.46	58.61 ± 32.39	11.94 ± 8.07	2.98 (2.61,3.78)	0.2 (0.1,0.4)	0.55 ± 0.09

*SF* serum ferritin, *UIBC* unsaturated iron-binding capacity, *SI* serum iron, *TRF* transferrin, *CRP* C-reactive protein, *α-AGP* α- acid glycoprotein

### Iron circulation rate

The iron circulation rates were calculated by the baseline figures (day 0) and the peak figures (the 2nd collection, ~day 20) of abundance curve according to the formula 3. Only 48 blood samples were obtained on the second collection, and they all contributed to the calculation of the iron circulation rates, while 2 of them were excluded as outliers. So, The results of 46 subjects, 15 in group 1 and 31 in group 2, were shown in Table 3. The average iron circulation rate of the 46 subjects was 80.4%, and there was no significant difference between the two groups (79.44% vs 80.86%, *P* > 0.05). The average weight (68.65 vs 59.72 kg, *P <* 0.05) and BMI (27.02 vs 23.38 kg/m^2^, *P <* 0.05) of group 1 were higher than that of group 2. The average age of 46 subjects was 29.79 y, with no significant difference between 2 groups (*P* > 0.05).

**Table 3:** The circulation rates of 46 subjects

group	n	age	weight (kg)	BMI (kg/m^2^)	circulation rate (%)
1	15	29.27 ± 4.33	68.65 ± 11.41	27.02 ± 4.16	79.44 ± 4.41
2	31	30.07 ± 4.19	59.72 ± 8.28	23.38 ± 3.10	80.86 ± 4.29
total	46	29.79 ± 4.21	62.63 ± 10.2	24.57 ± 3.84	80.40 ± 4.33
t		−0.591	3.023	3.328	−1.044
*P*		0.558	0.004	0.002	0.302

*BMI* body mass index

### Definition of the steady period

After consuming ^58^Fe, all subjects were followed up for more than 2 years. 11 subjects in group 2 with complete data of blood samples (day 0, 21, 39, 130, 253, 376, 499, 630, 810) were used to identify the steady period. The mean age of 11 subjects was 30, ranging from 22 to 39. The mean height, weight and BMI was 156.14 cm, 55.77 kg and 23 kg/cm^2^, respectively.

**Fig. 1:**
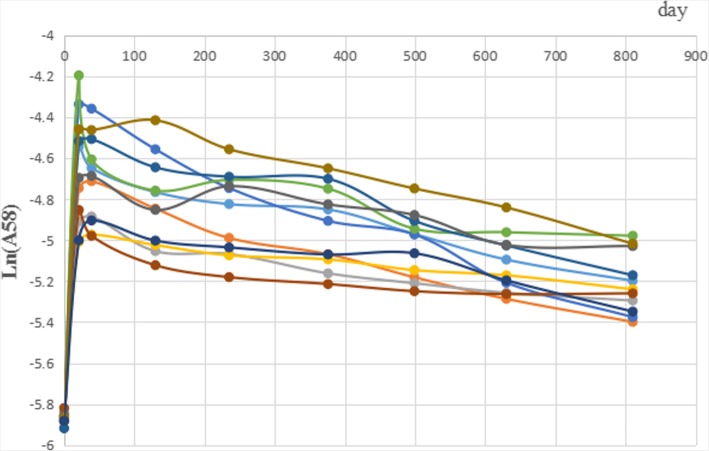
The abundance of ^58^Fe in blood

The abundance of ^58^Fe was log-transformed (natural logarithm) because it was exponentially changed after the steady period. The change of isotopic abundance (lnA (^58^Fe)) in blood are shown in Fig. 1. After ^58^Fe ingestion, the abundance increased to the peak and then decreased. While the RMANOVA by SAS software did not identify a significant turning point of the abundance curve. Combined with the figure and previous studies, the steady period was regarded to be started from day 376 [18–20].

### Physiological requirements for iron

After the steady period, blood samples from adjacent collection time can be used to calculate the physiological requirements for irons. 21 subjects, 6 in group 1 and 15 in group 2, with more than 2 sample collections per subject after the steady period were available for calculation. The mean physiological requirement for iron in 21 subjects calculated by formula 1 was 1.55 mg/d and 23.63 μg.kg^− 1^.d^− 1^ after body mass adjustment. Although body mass and BMI differed between the two groups (*P* < 0.05), there was no significant difference in physiological requirements for iron (1.74 vs 1.51 mg/d, *P* > 0.05) and that adjusted by body mass (22.46 vs 24.42 μg.kg^− 1^.d^− 1^, *P* > 0.05) (Table 4).

**Table 4:** Iron physiological requirements by formula calculation

group	n	age	weight (kg)	BMI (kg/m^2^)	iron physiological requirement (mg/d)	iron physiological requirements adjusted by weight (μg.kg^− 1^.d^− 1^)
1	6	32.50 ± 3.02	76.54 ± 10.58	30.00 ± 4.35	1.74 ± 0.64	22.46 ± 7.33
2	15	30.54 ± 4.88	60.35 ± 10.15	23.99 ± 3.93	1.50 ± 0.46	24.42 ± 8.08
total	21	31.16 ± 4.39	64.87 ± 12.79	25.67 ± 4.95	1.55 ± 0.52	23.63 ± 7.87
t	–	0.901	3.265	3.080	0.929	−0.515
*P*	–	0.380	0.004	0.006	0.365	0.612

*BMI* body mass index

**Fig. 2:**
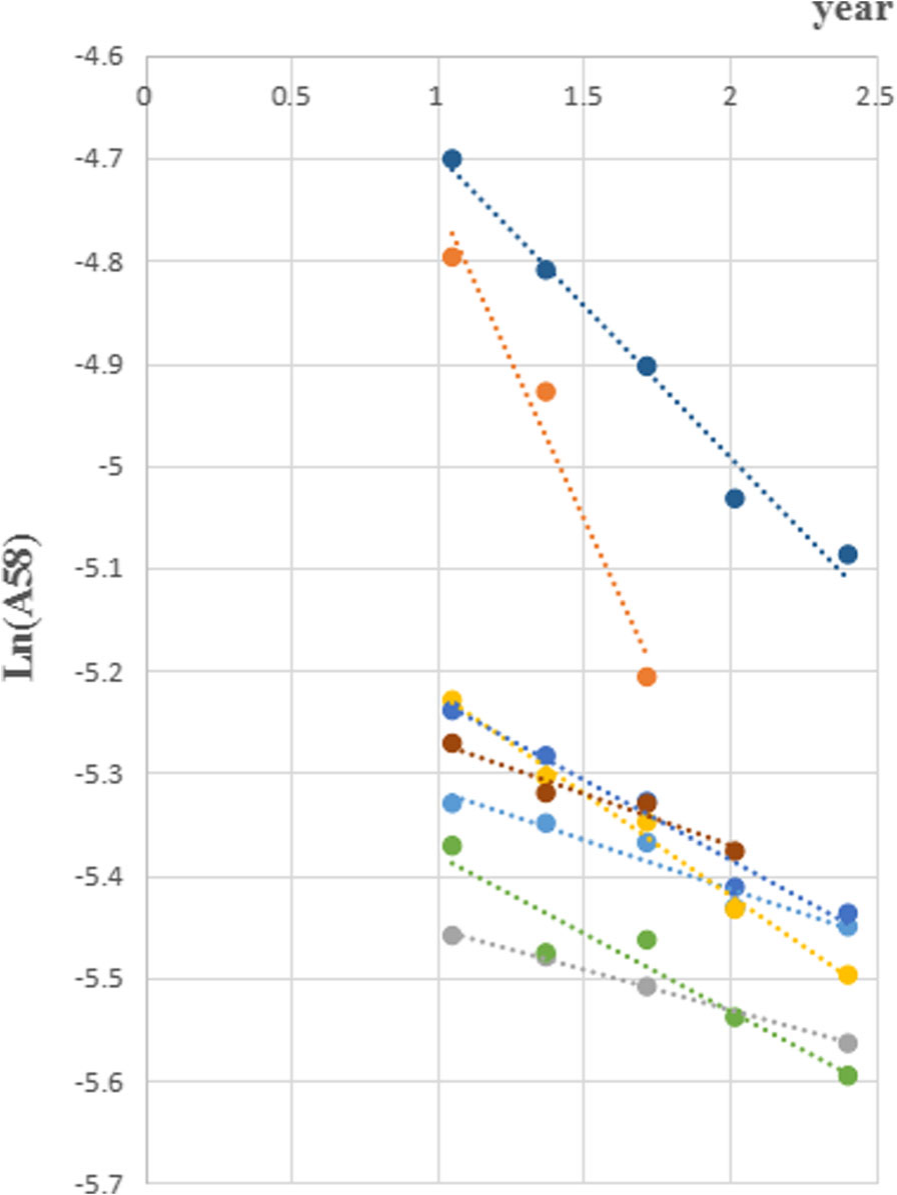
Liner regression in group 1

**Fig. 3:**
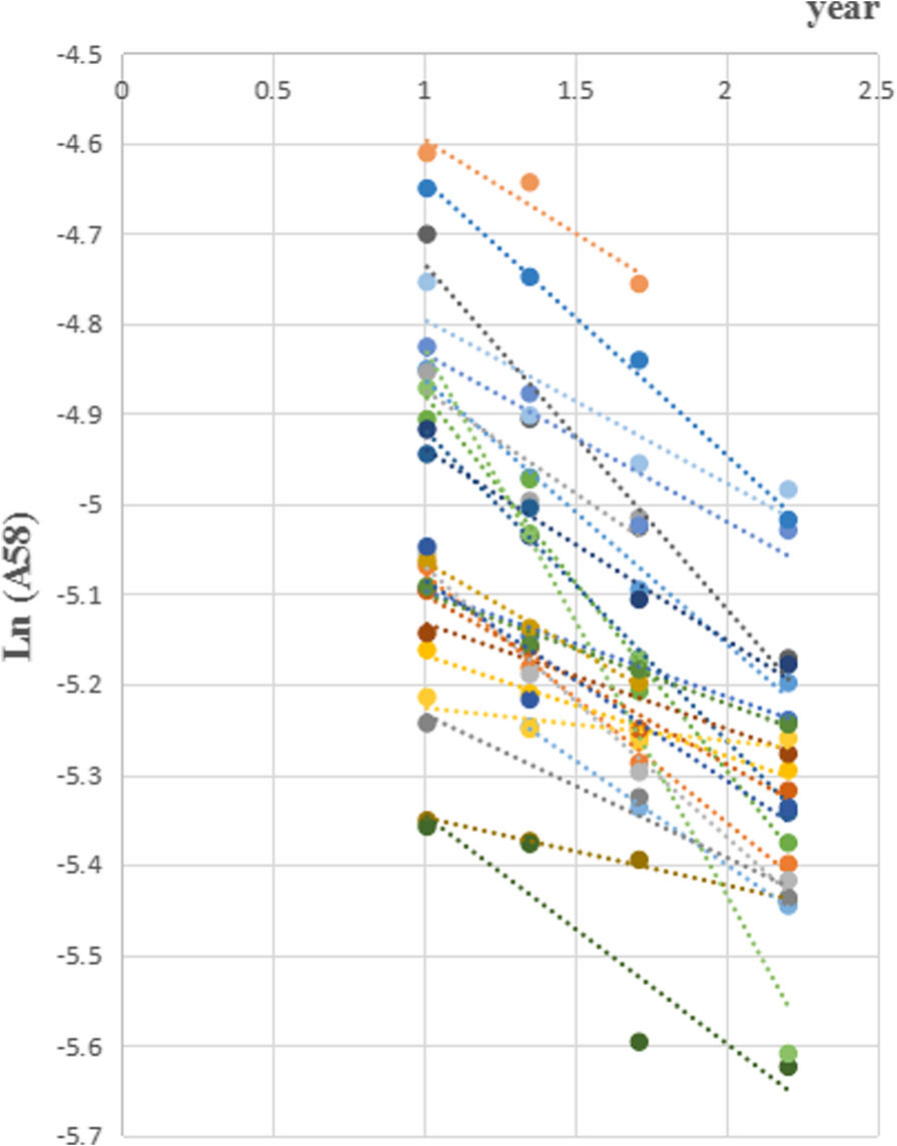
Liner regression in group 2

Meanwhile, the linear regression method was also used to obtain the physiological requirements. ^58^Fe abundance were logarithmic transformed and the regressed lines were drawn to obtain the annual iron loss rates (Figs. 2 and 3) The subjects whose R^2^ < 0.8 or R^2^ = 1 (only two time points could be used) of linear regression were excluded. 33 subjects, 8 in group 1 and 25 in group 2, were included in final analysis and shown in Table 5. The average physiological requirement for iron in these 33 subjects was 1.29 mg/d, 20.98 μg.kg^− 1^.d^− 1^, both of which showed no significant difference in 2 groups (*P* > 0.05). The median, lower quartile and upper quartile of R^2^ in 33 subjects were 0.9691 (0.9385,0.9861). The average annual iron loss rate was 22.71%. There was significant difference in weight (76.23 vs 60.49 kg, *P* < 0.05) and BMI (30.23 vs 24.06 kg/m^2^, *P* < 0.05) between 2 groups, and no significant difference in others (*P* > 0.05).

**Table 5:** Iron physiological requirements by liner regression

group	n	age	weight (kg)	BMI (kg/m^2^)	iron loss rate per year (%)	iron physiological requirement (mg/d)	iron physiological requirements adjusted by weight (μg.kg^−1^.d^−1^)
1	8	29.75 ± 5.20	76.23 ± 9.71	30.23 ± 3.26	21.16 ± 17.82	1.01 ± 0.66	14.60 ± 12.55
2	25	30.45 ± 4.67	60.49 ± 9.18	24.06 ± 3.92	23.20 ± 11.98	1.38 ± 0.70	23.01 ± 11.40
total	33	30.27 ± 4.76	64.31 ± 11.44	25.55 ± 4.59	22.71 ± 13.34	1.29 ± 0.70	20.98 ± 12.05
t	–	−0.353	4.165	4.017	−0.372	−1.317	−1.777
*P*	–	0.726	0.000	0.000	0.712	0.198	0.085

*BMI* body mass index

## Discussion

The iron circulation rate in women of reproductive age was 80.4%, which has no significant difference with the result 82.8% in our previous study [20], and was close to that in other studies. In the article published by our team in 2016, the ^58^Fe incorporation rate of males was 85% [25]. Hiroshi Saito et al. in 1964 reported that the average utilization radio of iron was 90, and 10% remained in tissues of 12 men [18]. Larsen L in 1975 reported that mean red cell utilization of absorbed ^59^Fe was 92.9% in adults [26]. The iron circulation rate was lower in infants, which was 75% in reported by Tondeur et al. [27] and 68% by McDonald et al. [28]. Different research subjects, including gender, age and research methods will lead to differences in results. In our study published in 2018 [20], the iron was infused and iron circulation rate was directly calculated by the iron in blood. However, in this study, ^58^Fe was taken orally and we couldn’t get accurate absorptivity of iron. So, we calculated the circulating iron (Fe_circ_), non-circulating active iron (Fe_nca_), and storage iron (Fe_stor_) in body according to the method by Fomon in 2005 [19] and then got the iron circulation rates.

In the related published studies, the determination of the steady period were mainly by observing the change curves of iron isotopes [18, 19]. We also used RMANOVA as before in this study, while no obvious turning point was found [20]. Finally, the steady period, about 1 year, was determined by observing when the abundance curve changed steadily and combined with results in other studies [18–20]. Therefore, when determining the steady period, the method should be appropriately chosen according to the data and make a comprehensive decision.

This study simultaneously used two methods to research the physiological requirement for iron. Linear regression is a classical method used in many studies [17, 19]. The regression coefficients in this study were all above 0.9, which ensured the reliability of the results. The formula calculation method, as an innovative method, had been introduced in our previous article [20]. When the weights of subject were unstable and the data were not insufficient for linear regression, it could be used for accurate calculation. In this study, the results of physiological requirements for iron by two methods showed no significant difference. The physiological requirement for iron in women of reproductive age was 1.29~1.55 mg/d, 20.98~23.63 μg.kg^− 1^.d^− 1^. In our previous study, the physiological requirement for iron in women was 1.1 mg/d, 20.69 μg.kg^− 1^.d^− 1^ [20]. Another study by Finch in 1959 reported 12 women in non-menstrual period, the physiological requirement for iron of which was 1.22 mg/d, 20 μg.kg^− 1^.d^− 1^ [21]. In our study, we researched the average results in a period of time (usually longer then 4 months), which means the total iron loss of women, including the basic loss and extra loss in menstrual periods. Although the methods and subjects varied in these studies, the results were relatively similar.

According to the physiological requirements for iron in women of reproductive age in this study, the iron DRIs could be estimated. EAR and RNI were calculated with reference weight of 56 kg, the absorption rate of 10% and the coefficient of variation of 15%. The results are shown in Table 6 and compared with the recommended value in Chinese (Chinese Dietary Reference Intake, 2013). The recommended value in 2013 were calculated by factorial calculation, in which the physiological requirement for iron was 1.47 mg/d, including the basal iron loss of 0.82 mg/d and the menstrual iron loss of 0.65 mg/d. The EAR and RNI obtained from the results in this study were slightly lower than the recommended values, mainly due to the different methodology and research subjects.

**Table 6:** Estimation of recommended intake of iron for women with child-bearing age

calculating method	n	weight (kg)	iron physiological requirement adjusted by weight (μg.kg^−1^.d^− 1^)	iron physiological requirement (mg/d)	reference weight (kg)	EAR (mg/d)	RNI (mg/d)
formula calculation method	21	64.87	23.63	1.55	56	13	18
linear regression method	33	64.31	20.98	1.29	56	11	15
recommended value		56	26.25	1.47	56	15	20

*EAR* estimated average requirement, *RNI* recommended intake

This study used two methods of data processing to research the physiological requirements for iron of women of productive age and compared the results. Due to the long time of follow-up, there were different degrees of dropping-out at various stages; as a result, the iron circulation rates and physiological requirements for iron were not derived from the exactly same subjects, which might affect the accuracy.

## Conclusion

In conclusion, the physiological requirements for iron in women of reproductive age calculated by 2 methods showed no significant difference, and were in accordance with other studies. The EAR and RNI calculated from these results were slightly lower than Chinese present recommended value. This study provides the basis for improving the methodology of physiological requirement for iron research and future revision of DRIs.

## Additional file

Additional file 1: Flow chart. (PNG 74 kb)

## Data Availability

The datasets used and/or analyzed during the current study are available from the corresponding author on reasonable request.

## Abbreviations

AAS: Atomic absorption spectroscopy
ANOVA: Analysis of Variance
CRP: C-reactive protein
DRI: Dietary reference intake
EAR: Estimated average requirement
IDA: Iron deficiency anemia
MC-ICP-MS: Multi-collector inductively-coupled plasma mass spectrometry
RNI: Recommended intake
RSD: Relative standard deviation
SF: Serum ferritin
SI: Serum iron
TRF: Transferrin
UIBC: Unsaturated iron-binding capacity
UL: Tolerable upper intake levels
α-AGP: α- acid glycoprotein
