# Supplementary figures and images for: Physiological requirements for iron in women of reproductive age assessed by the stable isotope tracer technique
Source: Nutr Metab (Lond). 2019 Aug 19;16:55. doi: 10.1186/s12986-019-0384-1 (PMC6701013; doi:10.1186/s12986-019-0384-1)

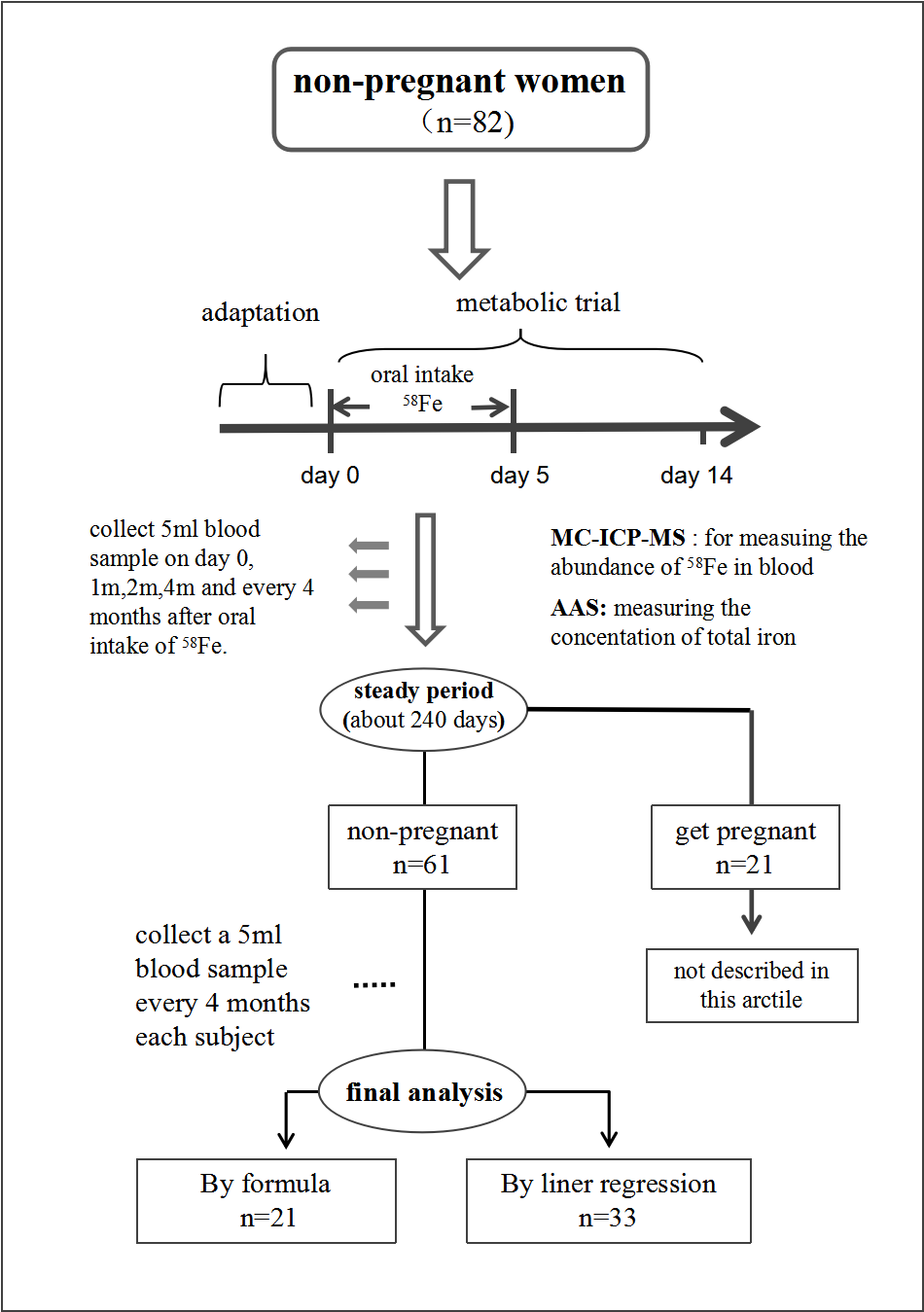

Supplement: Supplementary file 1 — Flow chart. (PNG 74 kb) [file 12986_2019_384_MOESM1_ESM.png]
